# Assessment of Rates of Suicide Risk Screening and Prevalence of Positive Screening Results Among US Veterans After Implementation of the Veterans Affairs Suicide Risk Identification Strategy

**DOI:** 10.1001/jamanetworkopen.2020.22531

**Published:** 2020-10-21

**Authors:** Nazanin Bahraini, Lisa A. Brenner, Catherine Barry, Trisha Hostetter, Janelle Keusch, Edward P. Post, Chad Kessler, Cliff Smith, Bridget B. Matarazzo

**Affiliations:** 1 Rocky Mountain Mental Illness Research, Education and Clinical Center, Rocky Mountain Regional Veterans Affairs (VA) Medical Center, Aurora, Colorado; 2 Department of Physical Medicine and Rehabilitation, University of Colorado Anschutz School of Medicine, Aurora; 3 Department of Neurology, University of Colorado Anschutz School of Medicine, Aurora; 4 Veterans Health Administration (VHA) Program Evaluation and Resource Center, Office of Mental Health and Suicide Prevention, Palo Alto, California; 5 VHA Center for Clinical Management Research, Ann Arbor, Michigan; 6 Ann Arbor VA Health Care System, Ann Arbor, Michigan; 7 Department of Internal Medicine, University of Michigan Medical School, Ann Arbor; 8 Durham VA Health Care System, Durham, North Carolina; 9 Department of Medicine, Duke University School of Medicine, Durham, North Carolina; 10 VHA Office of Mental Health and Suicide Prevention, Washington DC; 11 Department of Psychiatry, University of Colorado Anschutz School of Medicine, Aurora

## Abstract

**Importance:**

In 2018, the Veterans Health Administration (VHA) implemented the Veterans Affairs (VA) Suicide Risk Identification Strategy to improve the identification and management of suicide risk among veterans receiving VHA care.

**Objectives:**

To examine the prevalence of positive suicide screening results among veterans in ambulatory care and emergency departments (EDs) or urgent care clinics (UCCs) and to compare acuity of suicide risk among patients screened in these settings.

**Design, Setting, and Participants:**

This cross-sectional study used data from the VA’s Corporate Data Warehouse (CDW) to assess veterans with at least 1 ambulatory care visit (n = 4 101 685) or ED or UCC visit (n = 1 044 056) at 140 VHA medical centers from October 1, 2018, through September 30, 2019.

**Exposures:**

Standardized suicide risk screening and evaluation tools.

**Main Outcomes and Measures:**

One-year rate of suicide risk screening and evaluation, prevalence of positive primary and secondary suicide risk screening results, and levels of acute and chronic risk based on the VHA’s Comprehensive Suicide Risk Evaluation.

**Results:**

A total of 4 101 685 veterans in ambulatory care settings (mean [SD] age, 62.3 [16.4] years; 3 771 379 [91.9%] male; 2 996 974 [73.1%] White) and 1 044 056 veterans in ED or UCC settings (mean [SD] age, 59.2 [16.2] years; 932 319 [89.3%] male; 688 559 [66.0%] White) received the primary suicide screening. The prevalence of positive suicide screening results was 3.5% for primary screening and 0.4% for secondary screening in ambulatory care and 3.6% for primary screening and 2.1% in secondary screening for ED and UCC settings. Compared with veterans screened in ambulatory care, those screened in the ED or UCC were more likely to endorse suicidal ideation with intent (odds ratio [OR], 4.55; 95% CI, 4.37-4.74; *P* < .001), specific plan (OR, 3.16; 95% CI, 3.04-3.29; *P* < .001), and recent suicidal behavior (OR, 1.95; 95% CI, 1.87-2.03; *P* < .001) during secondary screening. Among the patients who received a Comprehensive Suicide Risk Evaluation, those in ED or UCC settings were more likely than those in ambulatory care settings to be at high acute risk (34.1% vs 8.5%; *P* < .001).

**Conclusions and Relevance:**

In this cross-sectional study, population-based suicide risk screening and evaluation in VHA ambulatory care and ED or UCC settings may help identify risk among patients who may not be receiving mental health treatment. Higher acuity of risk among veterans in ED or UCC settings compared with those in ambulatory care settings highlights the importance of scaling up implementation of brief evidence-based interventions in the ED or UCC to reduce suicidal behavior.

## Key Points

**Question:**

Are population-level suicide risk screening and evaluation feasible in Veterans Health Administration medical settings and do they identify patients at risk for suicide?

**Findings:**

In this cross-sectional study of more than 4 million US veterans screened in ambulatory care and emergency department settings during fiscal year 2019, the prevalence of suicidal ideation was 3.5%. Acuity of suicide risk was greater among patients screened in the emergency department than in ambulatory care.

**Meaning:**

Population-based suicide risk screening and evaluation in Veterans Health Administration medical settings may facilitate identification of risk among those who may not be receiving mental health treatment.

## Teaser

This cross-sectional study examines the prevalence of positive suicide screening results among veterans in ambulatory care and emergency departments or urgent care clinics and compare acuity of suicide risk among patients screened in these settings.

## Introduction

In 2016, the Joint Commission released a *Sentinel Event Alert*^1^ that shifted how health care systems approach the detection of suicide risk. This alert highlighted that a significant number of individuals who died by suicide were not receiving mental health care. Instead, in the months before their deaths, these individuals were seen in primary care, emergency department (ED), or other medical settings.^2,3,4^ These findings underscore the importance of suicide risk screening and evaluation across medical settings to identify patients with occult risk—those who may disclose suicidal thoughts and behaviors only if they are directly asked.^5^

During the last decade, the Veterans Health Administration (VHA) has made significant strides in suicide prevention. However, most of these efforts have focused on downstream interventions to reduce suicidal behavior among those already identified to be at high risk. Recognizing the need to implement more upstream efforts that are consistent with the *National Strategy for Preventing Veteran Suicide 2018-2028*,^6^ the VHA Office of Mental Health and Suicide Prevention established an interdisciplinary workgroup of experts to identify an evidence-informed approach to detect suicide risk among patients across VHA settings. This workgroup resulted in the development of the VA Suicide Risk Identification Strategy (Risk ID).^7^ Risk ID is an evidence-informed,^8^ enterprise-wide, 3-stage suicide risk screening and evaluation process: (1) primary screen: item 9 of the Patient Health Questionnaire 9 (PHQ-9),^9^ (2) secondary screen: Columbia Suicide Severity Rating Scale Screener (C-SSRS Screener),^10^ and (3) The VHA’s Comprehensive Suicide Risk Evaluation (CSRE). Individuals who screen positive at 1 level move on to the next level of screening or evaluation.

Risk ID moves beyond selective screening and evaluation and expands these practices to a broader population of patients, such as those presenting to ambulatory care (AC) settings and EDs or urgent care centers (UCCs). As part of Risk ID requirements, all veterans who are due for depression and/or posttraumatic stress disorder (PTSD) screening in AC settings also receive the primary screening for suicide risk. For most veterans, this occurs annually in primary care. Risk ID requirements in EDs and UCCs differ. Specifically, the primary screening is administered at each encounter during triage.

Emerging evidence suggests that suicide risk screening in AC and ED or UCC settings may provide critical opportunities to identify risk among patients who are not receiving or seeking mental health treatment.^5,11,12^ Although the feasibility and utility of screening in the ED or UCC setting and medical settings has been demonstrated in community hospitals,^5,11^ it has yet to be examined in the VHA, the country’s largest integrated health care system. In this article, we report findings from the first year of Risk ID implementation in AC as well as ED and UCC settings. Consistent with setting requirements, we describe the number of veterans who received the different stages of Risk ID as mandated. We also report the prevalence of positive primary and secondary screening results and levels of acute and chronic risk based on the CSRE. Last, we compare the acuity of suicide risk between patients presenting to AC settings as well as ED and UCC settings by examining differences in C-SSRS Screener responses and CSRE risk levels.

## Methods

Risk ID was implemented nationally throughout all VHA facilities (n = 140) in phases: primary screen (October 2018), secondary screen (December 2018), and the CSRE (November 2019). From October to December, practitioners could choose to follow up with the secondary screen (which was available in the electronic medical record) or some other risk assessment for those with a positive primary screening result. Implementation occurred across inpatient and outpatient settings. This study reports data specific to the AC setting and the ED and UCC settings during the first year of implementation (October 1, 2018, to September 30, 2019 [fiscal year 2019]). This study was exempted from review by the Colorado Institutional Review Board and approved by the VA Eastern Colorado Healthcare System Research and Development Committee. Informed consent was not required because the study was considered part of health care operations. A deidentified data set was used for the analyses. The study followed the Strengthening the Reporting of Observational Studies in Epidemiology (STROBE) reporting guidelines.^13^

### Settings and Patients

Patients were screened in AC settings based on the following criteria: having at least 1 nexus outpatient visit (eg, primary care, cardiology or hypertension, or mental health) and being eligible for depression and/or PTSD screening at the time of the visit (eg, did not have an existing diagnosis of depression, bipolar disorder, or PTSD). These patients represent approximately 77% of veterans seeking VHA care. Incomplete screening administrations and screens administered in Eds or UCCs were excluded.

The ED or UCC cohort eligibility included patients who had at least 1 visit to an ED or UCC. Screenings administered in non-ED or UCC settings or not documented in the national ED triage note template were excluded. Given the requirement to screen during every ED or UCC encounter, each unique visit was counted in the cohort and may have included multiple encounters by the same patient.

### Measures and Clinical Tools

#### PHQ-9 Item 9

The PHQ-9 is a reliable and validated measure of depression severity.^9^ Item 9 of the PHQ-9 asks about the presence of suicidal ideation during the past 2 weeks. Higher levels of suicidal ideation, indicated by item 9, have been associated with increased risk of suicide among VHA^14^ and non-VHA patients.^15^ In AC settings, item 9 was added to existing screens for depression (PHQ-2)^16^ and PTSD (Primary Care PTSD Screen).^17^ In the ED, item 9 was incorporated into the national ED triage template. Any response other than “none” was considered a positive primary screen and required the C-SSRS Screener to be completed on the same day (AC) or within 24 hours (ED or UCC).

#### C-SSRS Screener

The C-SSRS Screener^10^ is a 6-item measure that asks about the presence of suicidal ideation, method, intent, and plan during the last 30 days. Additional questions inquire about lifetime and recent suicidal behavior. A positive secondary screen was defined as a yes response to items 3, 4, 5, or 6b. Those with a positive secondary screening result were required to receive a same-day suicide risk evaluation (AC) or within 24 hours (ED or UCC). Reliability and validity of the original version of the C-SSRS Screener and the electronic version have been demonstrated.^10,18,19^

#### CSRE Tool

The CSRE is a clinical tool developed by the Risk ID workgroup. The CSRE includes evidence-based factors that are critical to assessing suicide risk and is consistent with recommendations outlined in the VA/US Department of Defense Clinical Practice Guideline for Assessment of Patients at Risk for Suicide^8^ as well as the Joint Commission element of performance for National Patient Safety Goal 15.01.01.^20^ These factors include assessment of suicidal ideation, plan, intent, suicidal behaviors, and risk and protective factors. Although the CSRE is a structured tool that details specific factors that should be assessed (eg, duration of suicidal ideation or history of suicide attempt), it is not a scripted assessment. Practitioners are encouraged to use their clinical expertise and knowledge of the veteran to flexibly conduct the evaluation and gather the required information. Practitioners then synthesize this information to stratify both acute (ie, minutes to days) and chronic (ie, longer than days) risk, which informs an individualized risk mitigation plan.^21,22^ Before national release, the CSRE went through rigorous pilot testing at multiple VHA facilities. Of note, the CSRE was not required during fiscal year 2019 but was available in the electronic health record for use. Facilities had the choice to use the CSRE or another method of suicide risk evaluation for those with a positive C-SSRS Screener result. Only findings specific to CSRE are reported in this article because other suicide risk evaluation methods could not be systematically captured.

### Data Sources

Primary outcomes include the number of patients receiving the different stages of Risk ID as intended. Secondary outcomes include prevalence of positive primary and secondary suicide screening results and levels of acute and chronic suicide risk. Data reported in this article, including patient demographics, were retrieved from the electronic health record via tables housed in the VA’s Corporate Data Warehouse.

In the AC cohort, the mental health instruments used were the item 9 of PHQ-2, item 9 of PC-PTSD-5, and C-SSRS Screener. Mental health survey administration data were used to identify the patient’s survey, administration date, clinical location, and survey results. The first survey administration at which the patient tested positive was selected as the index date; if no positive screening results were identified, the earliest survey administration was selected as the index date.

In the ED or UCC cohort, the mental health instruments used included item 9 (built into the national ED or UCC triage template), the C-SSRS Screener, and the CSRE. For item 9, health factor data identified survey results: positive, negative, or unable to respond. For the C-SSRS Screener, mental health survey administration data identified the patient’s survey, administration date, and survey results.

### Statistical Analysis

Consistent with setting requirements, completed screenings and 1-year rates of positive or negative screening results and CSRE risk levels were analyzed at the patient level in the AC cohort and at the encounter level in the ED or UCC cohort. Demographic information was analyzed at the patient level. Item responses for the C-SSRS Screener questions were compared between settings using a repeated-measures logistic regression with a generalized estimating equation to account for the correlation between repeated observations on an individual. The CSRE risk levels were compared between settings using χ^2^ tests, which assume independence. Given that differences between settings could be influenced by high-risk individuals presenting to EDs or UCCs on multiple occasions, the analysis was first conducted with repeat observations included. The analysis was then rerun with repeat observations removed to determine whether this accounted for the differences that were seen between the AC and ED or UCC cohorts. Post hoc pairwise comparisons were conducted for both cohorts. All analyses were conducted using SAS statistical software, version 9.4 (SAS Institute Inc). A 2-sided *P* < .05 was considered to be statistically significant.

## Results

A total of 4 101 685 US veterans in ambulatory care (mean [SD] age, 62.3 [16.4] years; 3 771 379 [91.9%] male; 2 996 974 [73.1%] White) and 1 044 056 veterans in ED or UCC settings (mean [SD] age, 59.2 [16.2] years; 932 319 [89.3%] male; 688 559 [66.0%] White) received the primary suicide screening (Table 1). The number of veterans who progressed through the different Risk ID stages are displayed in eFigure 1 (AC) and eFigure 2 (ED or UCC) in the Supplement.

**Table 1.** Patient Demographics for Patients Who Received the Primary Suicide Risk Screen by Setting, 2018-2019^a^

Demographic	Ambulatory care (n = 4 101 685)	ED or UCC (n = 1 044 056)
Age, mean (SD),	62.3 (16.4)	59.2 (16.2)
Sex		
Male	3 771 379 (91.9)	932 319 (89.3)
Female	330 303 (8.0)	111 736 (10.7)
Race/ethnicity		
White	2 996 974 (73.1)	688 559 (66.0)
Black or African American	695 039 (17.0)	266 708 (25.5)
Native Hawaiian or Other Pacific Islander	34 434 (0.8)	7960 (0.8)
Asian	46 254 (1.1)	8326 (0.8)
American Indian or Alaska Native	30 606 (0.8)	8576 (0.8)
Multirace	35 260 (0.9)	10 436 (1.0)
Missing	263 118 (6.4)	53 491 (5.1)

Abbreviations: ED, emergency department; UCC, urgent care clinic.

^a^ Data are presented as number (percentage) of patients unless otherwise indicated.

### Suicide Risk Screening and Evaluation: Uptake and Prevalence

In AC, 4 340 407 unique patients were eligible to receive the primary suicide screen as part of depression and/or PTSD screening. Of these, 4 101 685 patients (94.5%) were administered the primary suicide screen. Screening was conducted during 7 046 029 clinical encounters (ie, some patients in AC were screened during multiple encounters). A total of 5 559 649 screening encounters (78.9%) took place in primary care clinics. In the ED or UCC cohort, 1 988 946 of 2 500 564 encounters (79.5%) included administration of item 9 of the PHQ-9 during the visit. Table 2 lists prevalence of positive primary and secondary screening results by setting. Table 3 gives the distribution of acute and chronic risk levels for individuals receiving the CSRE by setting.

**Table 2.** Prevalence of Positive and Negative Screening Results by Setting, 2018-2019

Result	No. (%) of unique patients with item 9 response	
	AC (n = 4 101 685)	ED or UCC^a^ (n = 1 044 056)
Negative item 9^b^	3 959 053 (96.5)	1 025 175 (98.2)
Positive item 9^b^	142 632 (3.5)	37 761 (3.6)
No C-SSRS Screener^c^	45 406 (1.1)	6958 (0.7)
Negative C-SSRS Screener^d^	80 226 (2.0)	12 977 (1.2)
Positive C-SSRS Screener^d^	17 000 (0.4)	21 909 (2.1)

Abbreviations: AC, ambulatory care; C-SSRS Screener, Columbia Suicide Severity Rating Scale Screener; ED, emergency department; UCC, urgent care clinic.

^a^ In the ED or UCC cohort, categories are not mutually exclusive. For example, because unique individuals in the ED could have multiple encounters, they could have been counted in multiple categories if screening results differed during these encounters. In such cases, they would be counted only once in each category.

^b^ A total of 22 569 unique people in the ED or UCC cohort had a positive item 9 response and negative item 9 response on 2 separate encounters.

^c^ In the AC cohort, 1691 of those with no C-SSRS Screener result went from a positive item 9 response to a Comprehensive Suicide Risk Evaluation; in the ED or UCC cohort, 2346 of those with no C-SSRS Screener result went from a positive item 9 response to a Comprehensive Suicide Risk Evaluation.

^d^ A total of 2110 unique people in the ED or UCC cohort had a positive C-SSRS Screener result and a negative C-SSRS Screener result on 2 separate encounters.

**Table 3.** Acute and Chronic Suicide Risk Levels for Patients Who Completed a Veterans Affairs CSRE, by Clinical Setting, 2018-2019^a^

Risk level	AC (n = 8715 unique patients)	ED or UCC (n = 14 620 unique encounters)^b^
Acute		
High^c^	737 (8.5)	4987 (34.1)
Intermediate^d^	3560 (40.8)	6028 (41.2)
Low^c^	4370 (50.1)	3469 (23.7)
Missing	48 (0.6)	136 (0.9)
Chronic		
High^c^	828 (9.5)	3023 (20.7)
Intermediate^c^	4699 (53.9)	8444 (57.8)
Low^c^	3188 (36.6)	3153 (21.6)
Missing	0	0

Abbreviations: AC, ambulatory care; CSRE, Comprehensive Suicide Risk Evaluation; C-SSRS Screener, Columbia Suicide Severity Rating Scale Screener; ED, emergency department; UCC, urgent care clinic.

^a^ The CSRE numbers include the CSREs administered after a positive C-SSRS Screener result and CSREs administered directly after a positive item 9 result. The CSREs administered after a negative item 9 result or C-SSRS Screener result were not included in these comparisons.

^b^ Unique patients may be captured multiple times.

^c^ *P* < .001 (each risk level was compared with the 2 risk levels collapsed to calculate the *P* value).

^d^ *P* = .57 (each risk level was compared with the 2 risk levels collapsed to calculate the *P* value).

### Setting Specific Comparisons

The odds of endorsing specific items on the C-SSRS Screener (ie, yes response) were between 1.5 (ie, lifetime suicidal behavior) and 4.6 (ie, suicidal ideation with intent) times higher in the ED or UCC cohort compared with the AC cohort (Table 4). With respect to the CSRE, a high acute risk level was more likely to be assigned in the ED or UCC cohort compared with the AC cohort (34.1% vs 8.5%; *P* < .001). In contrast, a low acute risk level was more likely to be assigned in the AC cohort compared with the ED or UCC cohort (50.1% vs 23.7%; *P* < .001; ). For chronic risk, high and intermediate chronic risks were more likely to be assigned in the ED or UCC cohort compared with the AC cohort (high chronic risk, 20.7% vs 9.5%; intermediate chronic risk, 57.8%) (*P* < .001). Results remained significant when those presenting in both settings and those with multiple ED or UCC visits were removed.

**Table 4.** Item Response Breakdown for All Patients With Positive C-SSRS Screener Results by Setting, 2018-2019

C-SSRS Screener item	No. (%) of patients with positive responses		Odds ratio (ED or UCC vs AC) (95% CI)^a^
	AC (n = 17 000)	ED or UCC (n = 28 066)	
Thoughts of dying in past month	15 689 (92.3)	27 212 (97.0)	2.65 (2.43-2.89)
Suicidal ideation in past month	16 228 (95.5)	27 613 (98.4)	2.89 (2.57-3.25)
Suicidal ideation with consideration of method in past month	14 790 (87.0)	25 614 (91.3)	1.55 (1.46-1.65)
Suicidal ideation with intent in past month	7636 (44.9)	22 227 (79.2)	4.55 (4.37-4.74)
Suicidal ideation with specific plan in past month	5908 (34.8)	17 795 (63.4)	3.16 (3.04-3.29)
Suicidal ideation with specific plan and intent in past month	2938 (17.3)	13 780 (49.1)	4.43 (4.23-4.64)
Suicidal behavior during lifetime	9497 (55.9)	18 935 (67.5)	1.55 (1.50-1.62)
Suicidal behavior in past 3 mo	3995 (23.5)	10 765 (38.4)	1.95 (1.87-2.03)

Abbreviations: AC, ambulatory care; C-SSRS Screener, Columbia Suicide Severity Rating Scale Screener; ED, emergency department; UCC, urgent care clinic.

^a^ Odds ratios, 95% CIs, and *P* values are based on repeated-measures logistic regression (*P* < .001 for all).

## Discussion

In this cross-sectional study, the VHA implemented Risk ID as part of a comprehensive approach to suicide prevention; to date, it is the largest population-level suicide screening and evaluation program in the US. Findings indicate that suicide risk screening in AC and ED or UCC settings can be feasibly administered and may help identify veterans at risk for suicide. More than 4 million veterans in AC settings and more than 1 million veterans across 1.9 million EDs or UCCs underwent primary suicide screening as mandated. Furthermore, more than two-thirds of those with a positive primary screening result received a timely secondary screening. Although during this first year of implementation, when facilities could have used any method of suicide risk evaluation, the CSRE was still administered to more than 40% of patients with a positive secondary screening result. Such high rates of uptake in the first year of implementation are compelling in light of concerns raised about the feasibility and utility of standardized suicide risk screening and evaluation processes VHA-wide.^23^ Moreover, these findings are in line with an increasing body of evidence demonstrating that suicide screening and evaluation in medical settings is feasible, is effective, and can be accomplished without major disruptions to clinical work flows.^12^

Uptake of Risk ID was enhanced through multifaceted strategies, including education and training, technical assistance, and clinical decision support tools built into the electronic health record. For example, development of a national ED or UCC triage note template facilitated administration of item 9 of the PHQ-9 and allowed for warm handoffs to staff qualified to complete secondary screening. In AC settings, clinical reminders incorporated item 9 into existing screeners for depression and PTSD, both of which were already well integrated in routine practice.

The prevalence of positive item 9 results was similar in the ED or UCC and AC cohorts. Although this finding does not account for the patients with missing screening results, it indicates that a small yet significant number of veterans disclosed suicidal ideation when directly asked during what were largely considered non–mental health visits. Prevalence of suicidal ideation in VHA AC settings was higher than estimates of 1.4% obtained through other universal screening programs^5^ and community estimates of 3%.^24^ Although screening mostly took place in primary care and focused on patients without a diagnosis of depression or PTSD, some veterans may have been seeking care for other significant mental and behavioral health concerns (eg, anxiety or substance abuse). Prevalence of suicidal ideation in EDs or UCCs was slightly lower than estimates reported in other adult ED populations, which have ranged from 3.9% to 4.1%.^8,11^ Variation in screening measures used in EDs, time frame evaluated, and the criteria for positive screening results likely contribute to these differences.

More than two-thirds of patients in the ED or UCC cohort with a positive item 9 response advanced through all stages of Risk ID compared with one-fifth of those in the AC cohort. This finding points to some potentially important differences in the patients presenting to these settings. One consideration is that those screening positive for suicide risk in EDs or UCCs may be presenting with more acute risk. Furthermore, this greater level of acuity may not be readily apparent if relying on primary screening alone. Examination of responses on the C-SSRS Screener and CSRE risk levels support this notion. Specifically, the odds of endorsing each of the C-SSRS Screener items were significantly higher in the ED or UCC cohort compared with the AC cohort. This finding was particularly evident for items that reflect greater acuity, such as suicidal ideation with plan and intent. The CSRE risk levels also varied by setting such that patients in the ED or UCC cohort were more likely than those in the AC cohort to be categorized at high acute risk.

The greater acuity of suicide risk among patients in the ED or UCC cohort compared with those in the AC cohort supports national implementation of evidence-based suicide prevention programs, such as Safety Planning in the ED.^25,26,27^ Safety Planning in the ED provides additional support through safety planning and follow-up for patients at high or intermediate acute or chronic risk who are discharged home. This intervention builds on Risk ID with the aim of reducing future suicidal behavior and promoting treatment engagement. Although a smaller subset of individuals in the AC cohort were identified to be at high acute or chronic risk, it is notable that a significant number of individuals were still deemed to be at intermediate risk. Improving risk identification among patients presenting to AC settings, particularly primary care settings, may provide a critical opportunity to mitigate risk before the emergence of a suicidal crisis. A recent study^28^ found that almost half of VHA primary care patients who died by suicide did not have an antecedent mental health or substance use diagnosis. These patients were also more likely to die of firearm injury.^28^ Thus, in addition to suicide risk screening, identification and treatment of underlying mental and behavioral health conditions contributing to risk (eg, depression or substance abuse) are especially important, as is management of these conditions in primary care. Brief interventions focused on lethal means safety are also warranted given increased rates of suicide by firearm in this population.

First-year findings highlight the importance of ongoing evaluation to inform potential program modifications. This is a key component of any health care organization, such as the VHA, yet determining whether, when, and how evaluation data should be used to make program changes requires careful consideration of several factors, such as staffing, workflow processes, competing demands, and other policy requirements unique to specific settings. For example, recent changes to ED or UCC procedures to remove primary screening and begin with the C-SSRS Screener were based on a number of factors, including (1) low rates of ruling out those with positive item 9 screening results during secondary screening, (2) high correspondence of those scoring positive on item 9 and endorsing suicidal ideation on the C-SSRS Screener, (3) high uptake of secondary screening in EDs or UCCs, and (4) issues noted by the Joint Commission regarding the validity of item 9 when used as a single-item screener.

### Limitations

These analyses have limitations. First, uptake was examined as a national average, but performance across facilities is likely more variable. Understanding factors that contribute to variable uptake of Risk ID is important for tailoring implementation strategies to sites that are late adopters or experiencing unique implementation barriers. Along these lines, efforts are under way to examine whether an adaptive implementation strategy that adjusts the dose of implementation support based on facility performance effectively improves Risk ID uptake.^29^ Another limitation is the specific focus on AC and ED or UCC settings. Further evaluation of implementation across settings (eg, inpatient medicine) and whether these findings generalize to veterans not seeking VHA care is needed. Future research should also focus on understanding the impact of population-based suicide screening and evaluation on patient outcomes and suicide rates.

## Conclusions

Risk ID is a crucial step toward a unified strategy to improve the detection and management of suicide risk among all veterans presenting to the VHA for care. First-year findings indicate that population-based screening and evaluation in VHA AC and ED or UCC settings is not only feasible but also may also help identify risk among patients who for the most part may not be receiving or seeking mental health treatment. The higher acuity of risk among veterans presenting to ED or UCC compared with AC settings highlights the importance of scaling up implementation of brief evidence-based interventions designed for ED or UCC settings to promote treatment engagement and reduce suicidal behavior.^25,26,27^ Improving implementation of Risk ID across health care settings and identifying effective interventions for mitigating suicide risk that can be feasibly administered in primary care are important areas for further investigation

## Notes

Supplement. **eFigure 1.** Number and Percentages of Primary and Secondary Screens and CSREs Completed in Ambulatory Care, 2018-2019 **eFigure 2.** Number and Percentages of Primary and Secondary Screens and CSREs Completed in ED/UCC
