# Supplementary material for: Assessment of Rates of Suicide Risk Screening and Prevalence of Positive Screening Results Among US Veterans After Implementation of the Veterans Affairs Suicide Risk Identification Strategy
Source: JAMA Netw Open. 2020 Oct 21;3(10):e2022531. doi: 10.1001/jamanetworkopen.2020.22531 (PMC7578771; doi:10.1001/jamanetworkopen.2020.22531)
Supplement: Supplement. — eFigure 1. Number and Percentages of Primary and Secondary Screens and CSREs Completed in Ambulatory Care, 2018-2019 eFigure 2. Number and Percentages of Primary and Secondary Screens and CSREs Completed in ED/UCC [file jamanetwopen-e2022531-s001.pdf]

Supplementary Online Content

Bahraini N, Brenner LA, Barry C, et al. Assessment of rates of suicide risk screening and prevalence of positive screening results among US Veterans after implementation of the Veterans Affairs Suicide Risk Identification Strategy. *JAMA Netw Open*. 2020;3(10):e2022531. doi:10.1001/jamanetworkopen.2020.22531

eFigure 1. Number and Percentages of Primary and Secondary Screens and CSREs Completed in Ambulatory Care, 2018-2019

eFigure 2. Number and Percentages of Primary and Secondary Screens and CSREs Completed in ED/UCC

This supplementary material has been provided by the authors to give readers additional information about their work.

© 2020 Bahraini N et al. *JAMA Network Open.*

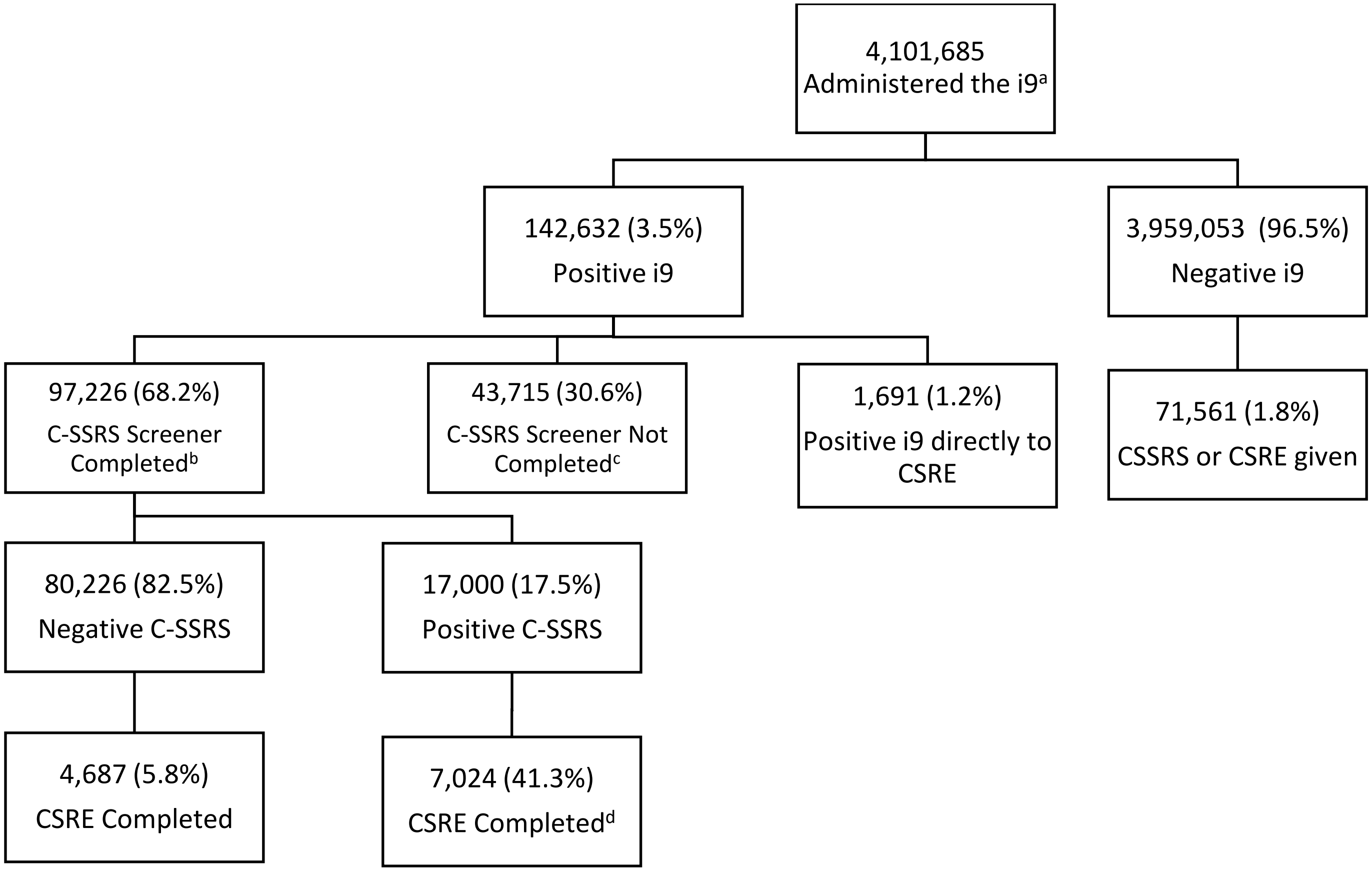

eFigure 1. Number and percentages of primary and secondary screens and CSREs completed in Ambulatory Care, 2018-2019

[a]94.5% of the 4,340,407 unique patients eligible for depression or PTSD screening. 238, 722 (5.5%) of patients eligible for depression or PTSD screening did not have a documented primary suicide risk screen. [b]Same day completion of C-SSRS Screener; [c] C-SSRS Screener Not Completed includes cases in which administration was not attempted as well as partial or incomplete administrations; [d]Same day completion of CSRE

© 2020 Bahraini N et al. *JAMA Network Open.*

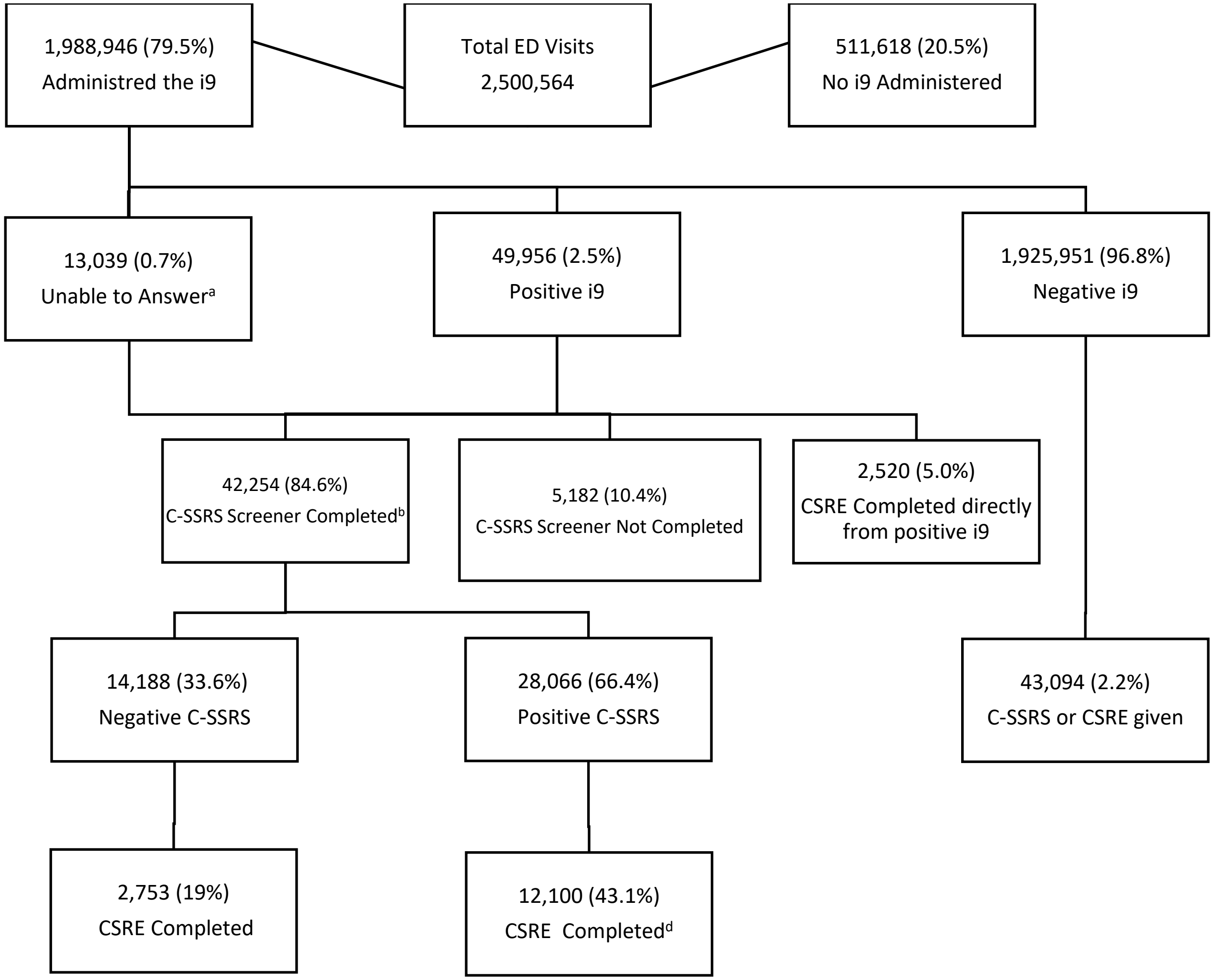

eFigure 2. Number and Percentages of Primary and Secondary Screens and CSREs Completed in ED/UCC

© 2020 Bahraini N et al. *JAMA Network Open.*
